# Bisphosphonates enhance EGFR-TKIs efficacy in advanced NSCLC patients with EGFR activating mutation: A retrospective study

**DOI:** 10.18632/oncotarget.5515

**Published:** 2015-11-27

**Authors:** Chu-Ying Huang, Li Wang, Cheng-Jun Feng, Ping Yu, Xiao-Hong Cai, Wen-Xiu Yao, Yong Xu, Xiao-Ke Liu, Wen-Jiang Zhu, Yan Wang, Jin Zhou, You Lu, Yong-Sheng Wang

**Affiliations:** ^1^ Department of Thoracic Oncology, Cancer Center, and State Key Laboratory of Biotherapy/Collaborative Innovation Center of Biotherapy, West China Hospital, Sichuan University, Chengdu, China; ^2^ Department of Oncology, The Second People's Hospital of Sichuan, Chengdu, China; ^3^ Department of Medical Oncology, Enshi Tujia and Miao Autonomous Prefecture Central Hospital, Hubei Province, China; ^4^ Department of Dermatology, Enshi Tujia and Miao Autonomous Prefecture Central Hospital, Hubei Province, China; ^5^ Department of Oncology, The First People's Hospital of Longquanyi District, Chengdu, China

**Keywords:** bisphosphonates, EGFR-TKIs, NSCLC, EGFR mutations, bone metastases

## Abstract

**Background:**

Bisphosphonates have exhibited anti-tumor activity in non-small cell lung cancer (NSCLC). We aimed to evaluate whether the combination of bisphosphonates with tyrosine kinase inhibitors of EGFR (EGFR-TKIs) could obtain a synergistic effect on advanced NSCLC patients with EGFR mutations.

**Methods:**

Between January 2008 and October 2013, 114 advanced EGFR mutations NSCLC patients who received EGFR-TKIs as first-line therapy were recruited from two cancer centers. Patients were separated into EGFR-TKIs alone or EGFR-TKIs plus bisphosphonates (combination) group. Median progression free survival (mPFS), median overall survival (mOS) distributions and survival curves were analyzed.

**Results:**

Among the 114 patients, 62 had bone metastases (19 patients treated with EGFR-TKIs, 43 patients treated with EGFR-TKIs + bisphosphonates). Median PFS and OS were significantly improved in combination group compared with EGFR-TKIs group (mPFS: 15.0 vs 7.3 months, *P* = 0.0017; mOS: 25.2 vs 10.4 months, *P* = 0.0015) in patients with bone metastases. Among the 71 patients (19 patients with bone metastases) treated with EGFR-TKIs alone, patients with bone metastases had poor survival prognosis (mPFS:7.3 vs 12.1 months, *P* = 0.0434; mOS:10.4 vs 22.0 months, *P* = 0.0036). The survival of patients with bone metastases who received EGFR-TKIs plus bisphosphonates therapy was non-inferior to patients without bone metastases treated with EGFR-TKIs alone (mPFS: 15.0 vs 12.1 months, *p* = 0.1871; mOS: 25.2 vs 22.0 months, *p* = 0.9798).

**Conclusions:**

Concomitant use of bisphosphonates and EGFR-TKIs improves therapeutic efficacy and brings survival benefits to NSCLC patients with EGFR mutation and bone metastases.

## INTRODUCTION

Lung cancer is the most common cancer worldwide [1]. Non-small-cell lung cancer (NSCLC) accounts for 85% of lung cancer and is often diagnosed at an advanced stage (inoperable stage IIIB or stage IV). For patients with advanced lung cancer, the incidences of bone metastases at diagnosis and at follow-up are approximately 30%-40%. Median overall survival time is <6 months and the 5-year survival rate is <5% [2]. Bone metastases may lead to skeletal-related events (SREs) such as bone pain, hypercalcemia, spinal cord compression, and pathologic fractures. Median survival time after the first SREs in patients with advanced lung cancer with bone metastases is only 4.1 months and SREs tend to have worse survival [3, 4].

Bisphosphonates are stable analogues of inorganic pyrophosphate that induce osteoclast apoptosis, inhibit bone resorption, osteoclast formation and recruitment [5]. Among the new-generation bisphosphonates, Zoledronic acid is efficient for the prevention of skeletal complications and generally recommended in patients with symptomatic bone metastases. Recently, preclinical data have shown that zoledronic acid might exert anti-tumor effects in NSCLC, including the inhibition of tumor cell proliferation, invasion, angiogenesis, and micrometastasis [6–10]. Clinical data also suggest that bisphosphonates may exhibit anti-tumor activity and prevent tumor progression of NSCLC [11, 12].

EGFR mutation is observed in 10% of the European NSCLC patients, and it is as high as 30–40% in East Asian patients. The objective response rate is about 71.2% when treated with EGFR-TKIs as the first-line therapy for the EGFR mutation advanced NSCLC [13], and the survival of patients with EGFR mutation is similar with EGFR wild type patients [14]. A retrospective clinical study found that the incidence of bone metastasis was similar in NSCLC patients with EGFR mutations compared to EGFR wild type patients [15]. Therefore, EGFR mutation NSCLC patients in the course of disease with bone metastasis are very common. Clinically, those patients with bone metastases and EGFR sensitive mutation of NSCLC, often used bisphosphonates and EGFR-TKIs concomitantly. However, whether the treatment effects could be improved by combination with bisphosphonates and EGFR-TKIs in these patients is still unknown. Two retrospective studies indicated that bisphosphonates combined with sunitinib/sorafenib could improve the response rate, PFS and OS in renal cell carcinoma with bone metastases [16, 17], which suggested that the combination of bisphosphonates and EGFR-TKIs probably also bring benefits to NSCLC. In the present study, we aimed to assess whether bisphosphonates can bring survival benefits to advanced NSCLC patients with bone metastases and treated with EGFR-TKIs.

## RESULTS

### Patient characteristics

One hundred and fourteen patients (mean ± SD: 59.05 ± 11.2, range 36–85, male 43.9%) with metastatic NSCLC (stage IV) received EGFR-TKIs as first-line treatment between 1st January 2008 and 31st October 2013 were included in this study. All patients harboring EGFR activating mutations were confirmed by ARMS-PCR or sequencing. In 114 patients, 73 (64.0%) were 19-del mutation and 41 (36.0%) were L858R mutation. The number of patients receiving gefitinib, erlotinib and icotinib treatment was 81, 16 and 17, respectively. In these 114 patients, 62 patients had bone metastases. Among these 62 patients, 43 were bisphosphonates users and 19 were nonusers due to oligo, small and asymptomatic bone metastases. In another 52 patients without bone metastases patients, none of them received bisphosphonates. Those patients who have received bisphosphonates, 35 were treated with zoledronic acid (4 mg monthly) and 8 with pamidronate (90 mg monthly). Therefore, among all the 114 patients, 71 were treated with EGFR-TKIs alone and 43 with EGFR-TKIs plus bisphosphonates. During EGFR-TKI treatment period, there was no one SREs occurrence in EGFR-TKIs alone group. In all patients, 109 had adenocarcinoma histology, 5 patients had squamous cell carcinoma. Nine of 114 (7.9%) patients had postoperative recurrent disease. All the distributions of clinic-pathologic characteristics were well balanced between the two treatment groups and were shown in Table 1.

**Table 1:** Patient characteristics

Patient characteristics	Only TKI (*n* = 52)	TKI +BPs (*n* = 43)	TKI alone(with bone metastases, *n* = 19)
Age-(year)			
Mean	59	59.2	58.6
Rang	38–85	36–81	38–85
Sex			
Male	21	20	9
Fenale	31	23	10
Histologic feature of tumor			
Adenocarcinoma	49	43	17
Squamas cell	3	0	2
EGFR statue			
L858R	17	16	8
19DEL	35	27	11
Disease stage			
IV	52	43	19
Time from diagnosis to start of TKI			
< 6mo	50	39	18
≥6 mo	2	4	1
Site of metastasis			
Brain	11	11	6
Liver	1	2	0
Bone	0	43	19
Sites of metastases ≥3	0	17	7
Suprarenal gland	3	3	2
Patient characteristics at start of TKI			
WHO performance status			
0–1	52	43	18
2–3	0	0	1
WC < 3.5 × 109	2	2	1
Neutrophils <2 × 109	1	1	0
Platelets <100 × 109	4	2	0
Haemoglobin <11.5 g/dl(woman), <13 g/dl(man)	16	16	9
LDH41.5x ULN	24	20	5
ALP	1	16	4

### EGFR-TKIs and bisphosphonates treatment efficacy

**Figure 1:**
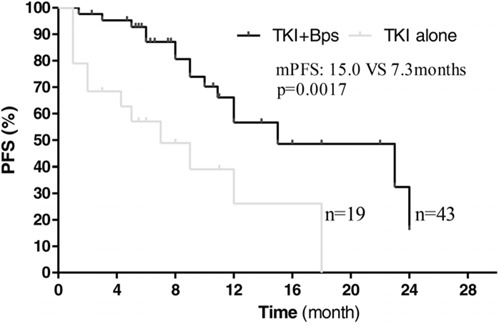
Kaplan–Meier curves showing progression-free survival, stratified by the use of bisphosphonates

**Figure 2:**
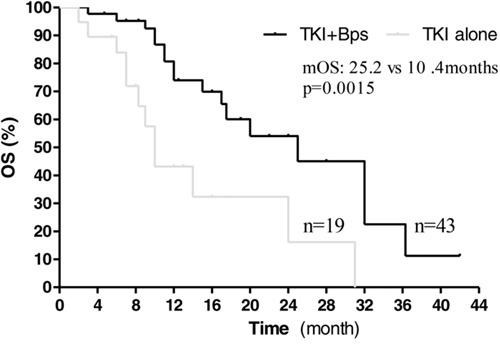
Kaplan–Meier curves showing overall survival, stratified by the use of bisphosphonates

Median follow-up time was 12 months (mean ± SD: 14 ± 8.6, range 1–72). All patients completed the treatment successfully, and none of them withdrew the treatment due to unacceptable adverse effects. Firstly, we investigated the effects of Bisphosphonates on these patients with both EGFR activating mutation and bone metastases. Among the 114 patients, 62 patients with bone metastases received EGFR-TKIs or EGFR-TKIs plus bisphosphonates as first-line therapy (19 patients treated with EGFR-TKIs alone, 43 patients treated with EGFR-TKIs plus bisphosphonates). The results showed that median PFS was significantly improved in the combination group compared to the EGFR-TKIs alone group (15.0 vs 7.3 months; HR 2.500, 95% CI: 2.022 to 2.978, *p* = 0.0017, Figure 1). Median OS was also significantly longer in the combination group than in the EGFR-TKIs alone group (25.2 vs 10.4 months; HR 2.143, 95% CI: 1.670 to 2.616, *p* = 0.00015, Figure 2). Therefore, among those patients with bone metastases, treatment with EGFR-TKIs plus bisphosphonates had superior efficacy compared to EGFR-TKIs treatment alone.

**Figure 3:**
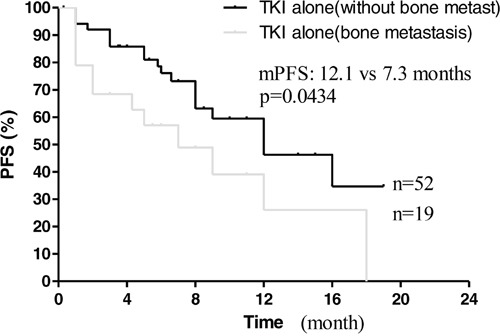
Kaplan–Meier curves for progression-free survival are shown for patients with bone metastases

**Figure 4:**
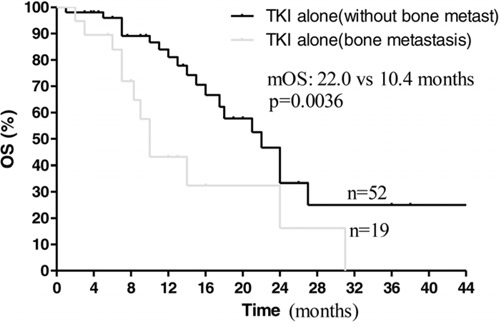
Kaplan–Meier curves for overall survival are shown for patients with bone metastases

Next, we compared the effect of EGFR-TKIs treatment alone on those patients with or without bone metastases. In 71 patients treated with EGFR-TKIs alone, there were 19 patients with bone metastases. The 19 patients received EGFR-TKIs treatment alone due to oligo and small bone metastases and without symptoms associated with bone metastases. Those patients finally did not suffer from SREs. However, those patients with bone metastases had worse survival (mPFS: 7.3 vs 12.1 months, *P* = 0.0434; mOS: 10.4 vs 22.0 months, *P* = 0.0036, Figure 3, 4), indicating bone metastases brought adverse effects and was a predictor for poor prognosis.

**Figure 5:**
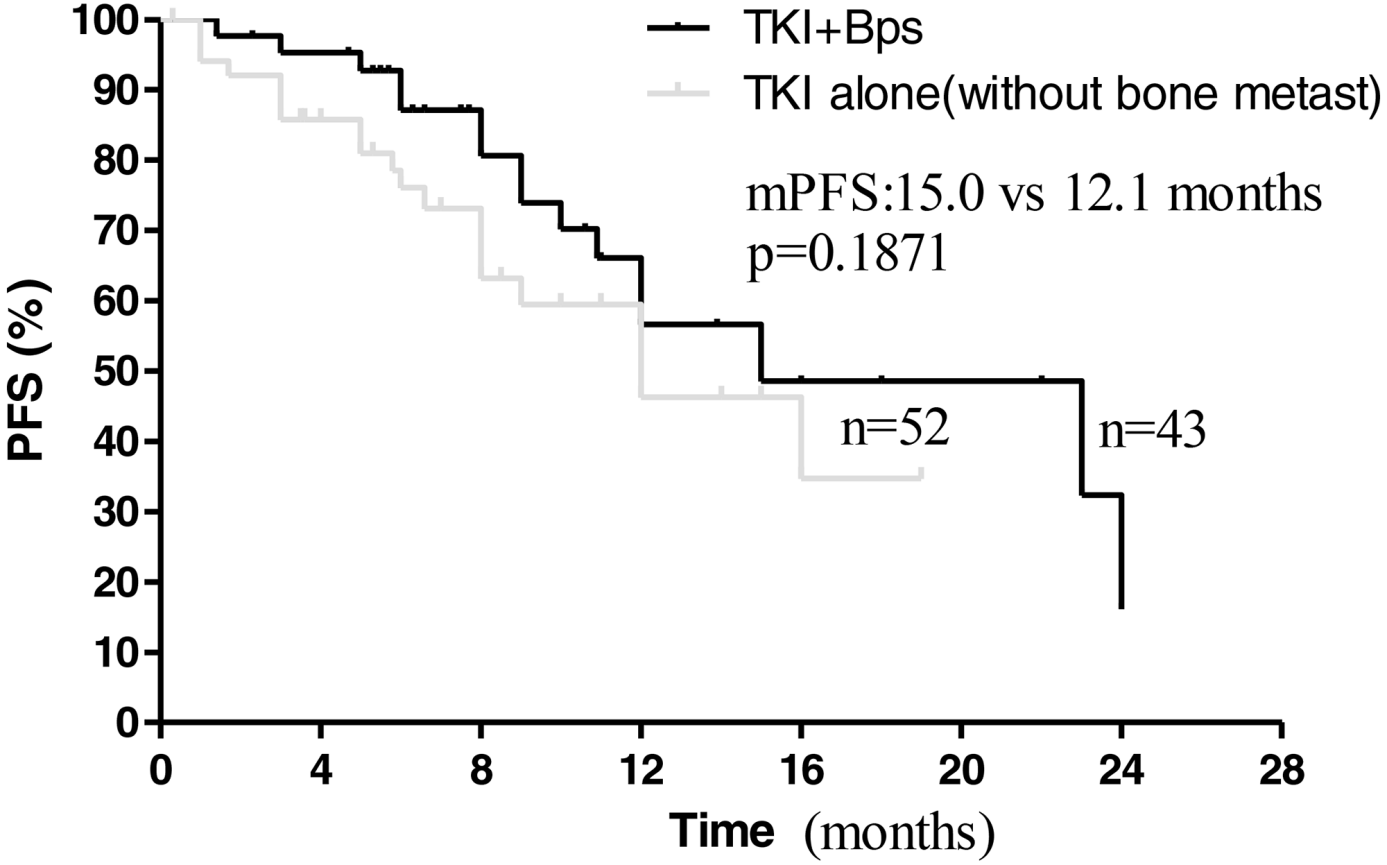
Kaplan–Meier curves for progression-free survival are shown for patients without bone metastases treated with TKI alone and patients treated with TKI+BPs

**Figure 6:**
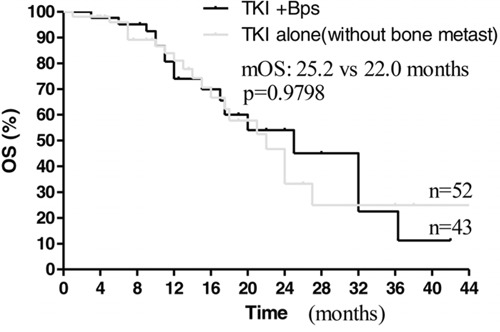
Kaplan–Meier curves for overall survival are shown for patients without bone metastases treated with TKI alone and patients treated with TKI+BPs

Subsequently, we compared the effect of EGFR-TKIs plus bisphosphonates to EGFR-TKIs alone treatment on those patients with or without bone metastases, respectively. Interestingly, among the 114 patients, 52 patients without bone metastases treated with EGFR-TKIs alone, 43 patients with bone metastases treated with EGFR-TKIs plus bisphosphonates as first-line therapy, median PFS time was 15.0 months in the EGFR-TKIs plus bisphosphonates group and 12.1 months in the EGFR-TKIs group (HR 1.250; 95% CI: 0.7358 to 1.764; *p* = 0.1871; Figure 5). Median OS time was 25.2 months in combination group and 22.0 months in EGFR-TKIs alone group (HR 1.136; 95% CI, 0.6166 to 1.656, *p* = 0.9798, Figure 6). The results suggested bisphosphonates treatment actually antagonized the adverse effects resulted from bone metastases.

In addition, the proportion of the 114 patients who responded during the treatment period (the best overall response of CR, PR, or SD) in the EGFR-TKIs alone group was lower than combination group (80.28% vs 90.69%, *P* = 0.299), but the difference was not statistically significant.

### Univariate analysis of factors associated with PFS and OS

Table 2 gives an overview of all previously described prognostic factors assessed by univariate analysis. The results showed that baseline hemoglobin (Hb) was associated with PFS (*p* = 0.012) and OS (*p* = 0.005). The Hb and alkaline phosphatase (ALP) in mPFS and mOS univariate analysis with a *P*-value <0.2 were included in multivariate model (Cox regression) to determine their independent effects.

**Table 2:** Univariate analysis

Parameter (number of patients)	Median PFS (month)	*P*	Median OS (month)	*P*
Bone metastases(53)	12	0.371	21	0.761
No bone metastases(62)	16		23	
Brain metastases(28)	16	0.39	13	0.789
No brain metastases(86)	12		22	
Hb <11.5 g/dl(woman), < 13 g/dl(man)(41)	9	0.012	18	0.005
Hb nomal(73)	16		24	
PLT <100×10^9/L(10)	10	0.807	16	0.164
PLT nomal(104)	15		23	
ALP >160 IU/L(21)	12	0.21	15	0.134
ALP nomal(93)	15		24	
LDH >220 IU/L(49)	15	0.405	25	0.582
LDH nomal(65)	12		20	
WC >10×10^9/L(13)	12	0.397	21	0.924
WC nomal(101)	16		23	
Gefetinib(81)	12	0.628	22	0.272
Erolotinib(16)	12		20	
Icotinib(17)	12		21	
19DEL(73)	10	0.48	24	0.43
L858R(41)	10		20	

### Multivariate analysis of factors associated with PFS and OS

In multivariate analysis (Table 3), bisphosphonate administration, baseline hemoglobin and baseline alkaline phosphatase were independently linked to PFS and OS.

**Table 3:** Multivariate analysis

	*P*-value	Hazard ratio	95% CI
For PFS			
Bisphosphonate administration or not	0.002	3.955	1.65–9.483
Baseline hemoglobin	0.037	0.515	0.275–0.962
Baseline alkaline phosphatase	0.005	0.265	0.105–0.666
For OS			
Bisphosphonate administration or not	0.004	3.566	1.489–8.540
Baseline hemoglobin	0.005	0.379	0.192–0.748
Baseline alkaline phosphatase	0.004	0.234	0.088–0.626

### Drug toxicity

Adverse events occurring in the treatment groups were listed in Table 4. All adverse events shown in Table 4 are acceptable. The most common adverse events in EGFR-TKIs group were skin rash followed by diarrhea, cutaneous pruritus and dry skin. By contrast, the most common adverse events in EGFR-TKIs plus bisphosphonates group were skin rash, cutaneous pruritus, diarrhea and liver dysfunction. There was no occurrence of jaw osteonecrosis. Our findings suggest that the combined treatment of EGFR-TKIs and bisphosphonates does not increase the side effects of EGFR-TKIs.

**Table 4:** Treatment-related adverse events

Patients with treatment-related adverse	TKI alone	TKI+BPs
Rash	12	4
Pruritus	2	3
dry skin	1	1
Diarrhoea	2	0

## DISCUSSION

Lung cancer is the most common cancer worldwide [1] and approximately 30%-40% patients with advanced NSCLC develop bone metastases [2]. Bisphosphonates are the most commonly used for the treatment of skeletal complications associated with bone metastases and have been proven to significantly delay the median time to first SRE and reduce the risk of skeletal complications among patients with bone metastases from lung cancer and other solid tumors [11, 18, 19]. In addition to the established SRE-prevention benefits, previous clinical studies also suggested that bisphosphonates therapy could provide survival benefits to NSCLC and other tumors patients with bone metastases [20–22]. Here, our investigation showed the effects of treatment with EGFR-TKIs plus bisphosphonates were better than EGFR-TKIs alone in those NSCLC patients with EGFR mutation and bone metastases. Among 62 patients with bone metastases, 19 patients were treated with EGFR-TKIs alone due to oligo, small and asymptomatic bone metastases. PFS and OS were significantly improved in the EGFR-TKIs plus bisphosphonates group compared with the EGFR-TKIs group. Multivariate analysis also showed bisphosphonate administration associated with improved PFS and OS. Therefore, for patients with single, small or asymptomatic metastatic bone lesions, our study supported bisphosphonates therapy should be used as early as possible.

During the EGFR-TKIs treatment course, there was no SREs occurrence in those patients with bone metastases. Therefore, we speculated that the prolonged PFS and OS with EGFR-TKIs plus Bisphosphonates treatment did not result from SRE-prevention benefits of bisphosphonates. Currently, serial preclinical studies have confirmed that bisphosphonates have direct antitumor effects on a variety of tumors, including NSCLC [23–25]. The mechanism includes regulating cell cycle, inducing tumor cells apoptosis, inhibiting tumor cell invasion and metastasis, affecting tumors molecular signal pathways, regulating immune microenvironment and anti-angiogenesis [24–29]. However, there are two researches studied whether bisphosphonates treatment NSCLC without bone metastasis could improve survival [30, 31]. One randomized, phase III study evaluated whether zoledronic acid could delay disease progression or recurrence in patients with controlled stage IIIA/B NSCLC after first-line therapy [30]. Another randomized, phase 2 study evaluated the ability of zoledronic acid to augment the cytotoxic effects of the combination of docetaxel/carboplatin and to delay disease progression in patients with inoperable stage IIIB or IV NSCLC [31]. In those two studies, zoledronic acid treatment did not improve the survival. Furthermore, even in a clinical trial in NSCLC patients with bone metastases, the combination of bisphosphonates and docetaxel also failed to improve the survival when compared with docetaxel alone [32]. These investigations suggested that clinical effects of bisphosphonates treatment alone or combining with chemotherapy on NSCLC patients are limited.

Two preclinical studies showed that combined treatment with gefitinib and zoledronic acid produced a tumor-suppressing effect significantly, which was more effective than the individual antitumor effect of gefitinib or zoledronic acid *in vitro* and *in vivo* [10, 33]. One study showed that gefitinib could inhibit ERK1/2 and Akt protein phosphorylation, result in tumor cells arrest in G1 phase and induce apoptosis [10]. Zoledronic acid could not only inhibit ERK1/2 and Akt activating in NSCLC, but it also could inhibit STAT3 activating in other tumors. Another study showed that zoledronic acid plus gefitinib could synergistically inhibit ERK1/2, Akt and STAT3 activating, which were the major signaling pathways of EGFR [33]. As known, re-activations of Akt and STAT have proven to be important for EGFR-TKIs resistance [34, 35]. The effects of bisphosphonates on downstream signaling of EGFR might bring potential clinical benefits for NSCLC patients with EGFR mutation and who received EGFR-TKIs treatment. In this study, our data revealed that EGFR-TKIs plus bisphosphonates indeed prolonged PFS and OS in EGFR mutation NSCLC patients who received EGFR-TKIs as the first-line therapy. There was other clinical evidence of survival benefits treated with bisphosphonates and EGFR-TKIs in some malignancies with bone metastases [16, 17]. A multicentre retrospective study indicated that bisphosphonates combined with sunitinib improved the response rate, PFS and OS in renal cell carcinoma with bone metastases [16]. Another retrospective study of 76 patients with metastatic renal cell carcinoma revealed that concomitant use of bisphosphonates and EGFR-TKIs in renal cell carcinoma patients with bone metastases probably improved treatment efficacy [17]. Therefore, we considered that bisphosphonates actually could enhance, even sensitize the effects of EGFR-TKIs on NSCLC patients with EGFR mutation. The effects of bisphosphonates on signaling of EGFR-TKIs resistance are speculated to be different from chemotherapy resistance, this may account for the enhanced antitumor effects and prolonged PFS and OS.

Our findings also showed that bone metastasis was an independent predictor of poor prognosis for advanced NSCLC patients with EGFR mutations received EGFR-TKIs treatment. Among these patients treated with EGFR-TKIs alone, significant difference of PFS and OS was observed between with and without bone metastases (Figure 3 and 4). Multivariate analysis also showed ALP associated with PFS and OS. As known, ALP level is closely related to malignant tumor with bone metastases [16, 17]. On the other hand, there was no prognostic difference observed between NSCLC patients with bone metastases received EGFR-TKIs plus bisphosphonates and those without bone metastasis treated with EGFR-TKIs alone (Figure 5, 6). This suggested that bisphosphonates treatment actually countered the adverse effects from bone metastases and brought survival benefits in EGFR mutation NSCLC patients with bone metastases. So far, there are no clinical data to answer whether bisphosphonates could enhance the effects of EGFR-TKIs in EGFR mutation patients without bone metastases. Our data may support further pre-clinical and clinical investigations.

We have to admit that our study has some limitations. First, in this retrospective clinical study, we are unable to exclude all the possibility of unequal distribution of unidentified clinic-pathologic parameters in each group. In addition, the number of cases in each group is relatively small, which might bias the results. Second, percentage of EGFR 19-del mutation (64%) is higher than L858R mutation in exon 21 (36%) in this study. As we know, 19-del mutation and L858R mutation each accounts for about 40–45% in all EGFR mutations of NSCLC [36, 37]. Previous study found that, 19-del mutation is more sensitive to EGFR-TKIs compared with L858R mutation [38, 39]. Therefore, different composing of cases may affect the results. Third, this study included 5 patients with squamous cell carcinoma, they all received EGFR-TKIs monotherapy. Previous studies have found that, even with EGFR mutations in squamous cell carcinoma, it is still the inferior efficiency treatment with EGFR-TKIs than adenocarcinoma with EGFR mutations [40]. Actually, we analyzed our data and get similar results when excluding the five patients (Supplementary Figure 1–6, S1–S6). Finally, bisphosphonates used in this study were mostly zoledronic acid, while treatment using other bisphosphonates were rare. Therefore, whether other bisphosphonates could improve patients survival combined with EGFR-TKIs needs further investigation.

In summary, our study confirmed that bisphosphonates combined with EGFR-TKIs prolonged PFS and OS of NSCLC patients with EGFR mutations and bone metastases. Our study also suggests bisphosphonates should be given as early as possible to the patients even with oligo bone metastases, and the enhanced effects of bisphosphonates on EGFR-TKIs may deserve to be investigated in further clinical trials.

## PATIENTS AND METHODS

### Patient selection

This was a retrospective study. The primary objective was to assess the impact of concomitant bisphosphonates on progression-free survival (PFS) under treatment with EGFR-TKIs. PFS was defined as the lapse of time between the start of EGFR-TKIs therapy and progressive disease under the targeted therapy or death. The secondary objective was to assess the impact of treatment with bisphosphonates on overall survival (OS) in such patients. OS was defined as the lapse of time between the start of targeted therapy and death of any cause.

One hundred and fourteen metastatic NSCLC patients (stage IV) with EGFR mutations were treated with EGFR-TKIs between 1st January 2008 and 31st October 2013, in two cancer centers (West China Hospital of Sichuan University and the Second People's Hospital of Sichuan, Sichuan province, China). Patients were eligible for inclusion in this study if they were 18 years of age or older, had been diagnosed as advanced NSCLC (stage IV), contained EGFR activating mutation, and received first-line EGFR-TKIs treatment, at least 1 measurable tumor lesions as evaluated by imaging detection. Patients who stopped EGFR-TKIs before completing 4 weeks of EGFR-TKIs treatment were excluded.

### EGFR-TKIs and bisphosphonates treatment

All patients received EGFR-TKIs treatment, including Gefitinib, Icotinib and Erlotinib. Treatment was continued until the evidence of disease progression, unacceptable adverse events or death. The decision whether to start bisphosphonates in those with bone metastases NSCLC patients were at the discretion of the treating physician. Bisphosphonates were administrated every 3–4 weeks according to the guidelines. Patients with concomitant bisphosphonates were defined as patients who started bisphosphonates together with EGFR-TKIs or who received them before and until the beginning or during the treatment with first-line EGFR-TKIs.

### Disease assessments

Follow-up time was defined as the time from EGFR-TKIs treatment initiation to 31st October 2013. During the treatment, all patients underwent chest CT-scan every 2–3 months. Bone metastases assessments were detected by CT scan, MRI and/or bone scintigraphy. Tumor response was defined according to the Response Evaluation Criteria in Solid Tumors 1.1 (RECIST 1.1) criteria. Treatment associated toxicity was evaluated according to the National Cancer Institute Common Terminology Criteria for Adverse Events (NCI-CTCAE) version 4.0.

### Statistical analysis

Progression-free survival and OS distributions were estimated using the Kaplan–Meier method and survival curves were compared with log-rank test. Univariate and multivariate Cox proportional hazards regression analyses were performed to determine independent prognostic factors for disease survival. All the variables with significant association in univariate analysis (by Kaplan–Meier with a *P*-value < 0.2) were included in multivariate model (Cox regression) to determine their independent effects. The relative risk (RR) and 95% confidence intervals (CI) were calculated. The value of p less than 0.05 was considered statistically significant. Data were analysed using the SPSS 16.0 (SPSS Inc., Chicago, Illinois) software package.

## SUPPLEMENTARY MATERIAL FIGURES


