# Supplementary material for: Bisphosphonates enhance EGFR-TKIs efficacy in advanced NSCLC patients with EGFR activating mutation: A retrospective study
Source: Oncotarget. 2015 Nov 27;7(41):66480–90. doi: 10.18632/oncotarget.5515 (PMC5341815; doi:10.18632/oncotarget.5515)
Supplement: Supplementary file 1 [file oncotarget-07-66480-s001.pdf]

Bisphosphonates enhance EGFR-TKIs efficacy in advanced NSCLC patients with EGFR activating mutation: A retrospective study

SUPPLEMENTARY FIGURES

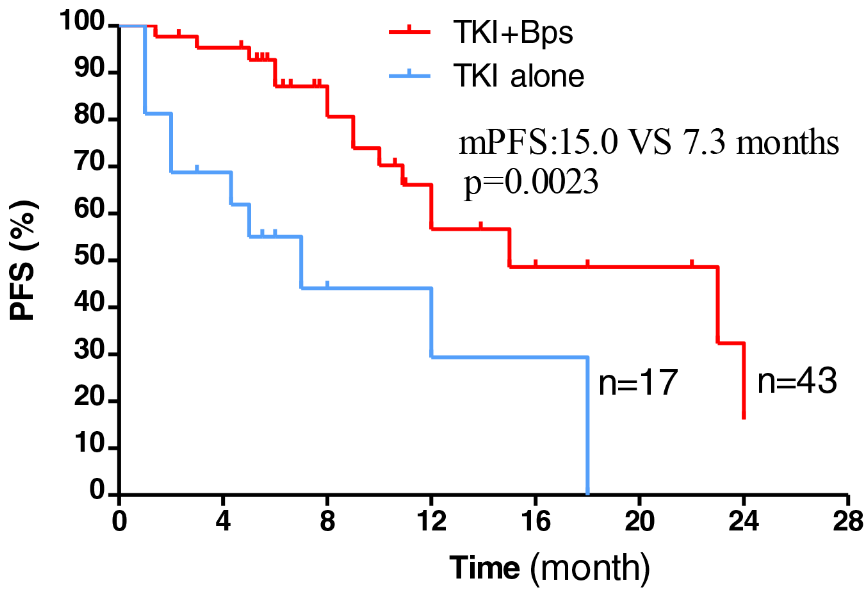

Supplementary Figure S1: Kaplan–Meier curves showing progression-free survival, stratified by the use of bisphosphonates.

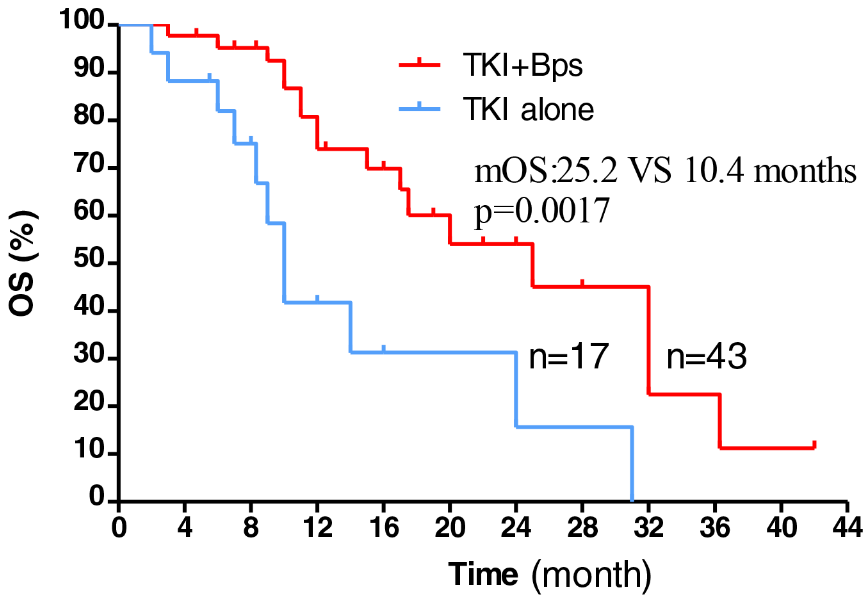

Supplementary Figure S2: Kaplan–Meier curves showing overall survival, stratified by the use of bisphosphonates.

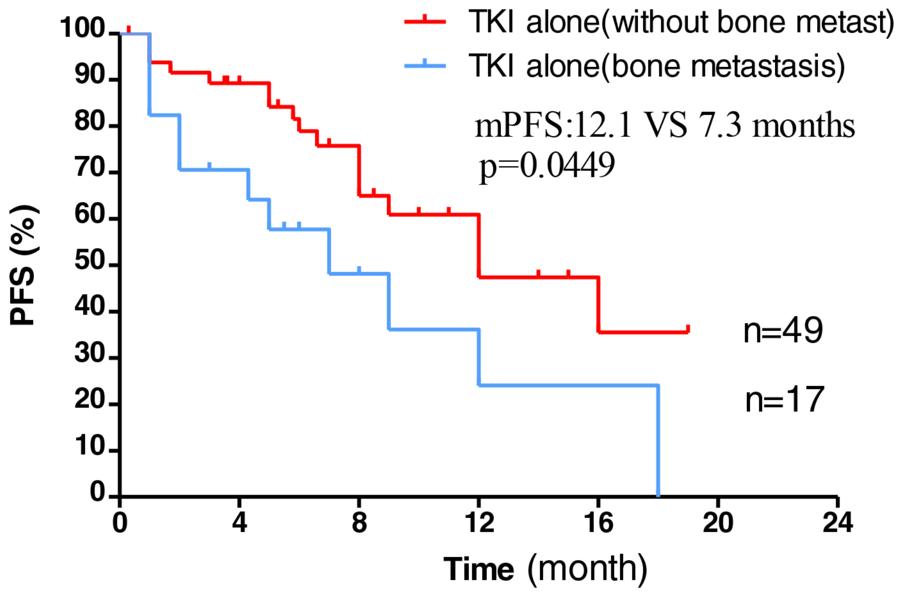

Supplementary Figure S3: Kaplan–Meier curves for progression-free survival are shown for patients with bone metastases.

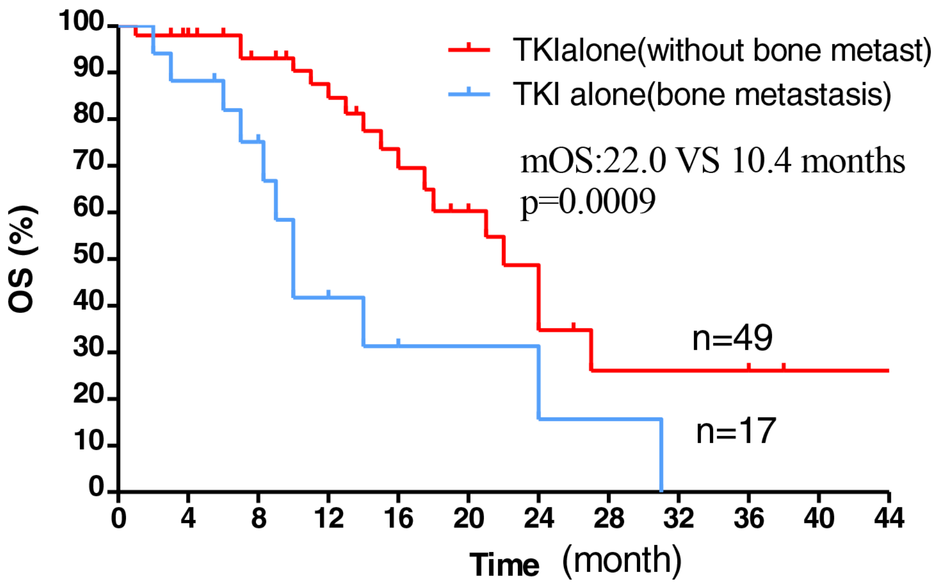

Supplementary Figure S4: Kaplan–Meier curves for overall survival are shown for patients with bone metastases.

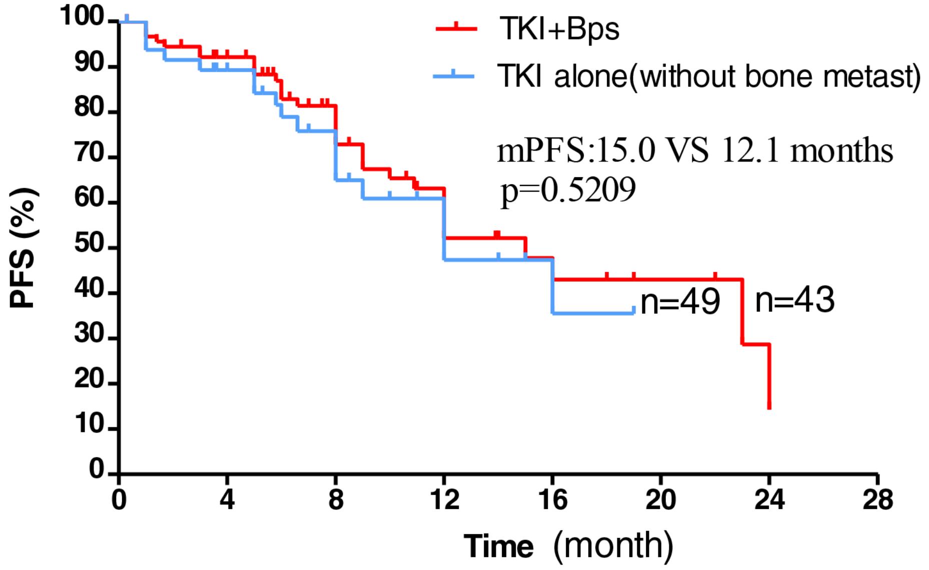

Supplementary Figure S5: Kaplan–Meier curves for progression-free survival are shown for patients without bone metastases treated with TKI alone and patients treated with TKI+BPs.

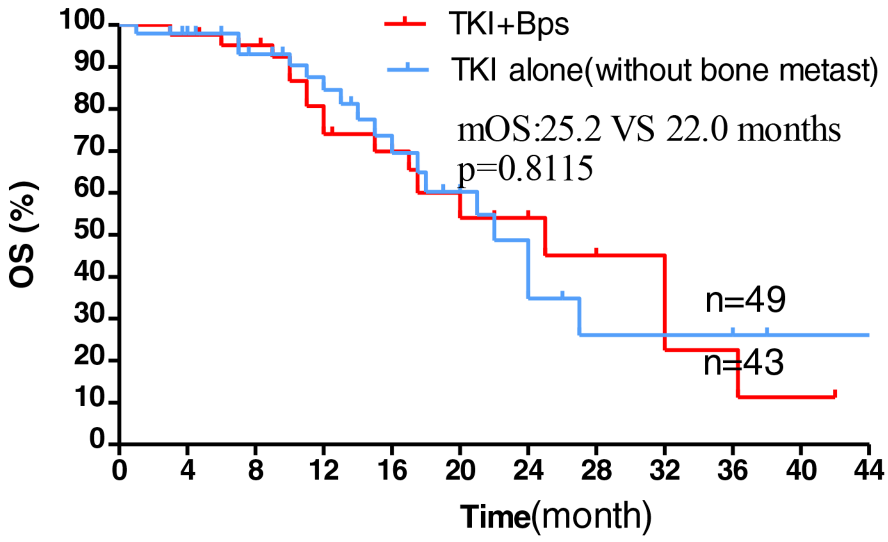

Supplementary Figure S6: Kaplan–Meier curves for overall survival are shown for patients without bone metastases treated with TKI alone and patients treated with TKI+BPs.
